# Association between eating speed and atherosclerosis in relation to growth differentiation factor-15 levels in older individuals in a cross-sectional study

**DOI:** 10.1038/s41598-024-67187-3

**Published:** 2024-07-17

**Authors:** Yuji Shimizu, Shin-Ya Kawashiri, Yuko Noguchi, Nagisa Sasaki, Mutsumi Matsuyama, Seiko Nakamichi, Kazuhiko Arima, Yasuhiro Nagata, Takahiro Maeda, Naomi Hayashida

**Affiliations:** 1 Epidemiology Section, Division of Public Health, Osaka Institute of Public Health, Osaka, 537-0025 Japan; 2 Department of General Medicine, Nagasaki University Graduate School of Biomedical Sciences, Nagasaki, 852-8523 Japan; 3 Department of Community Medicine, Nagasaki University Graduate School of Biomedical Sciences, Nagasaki, 852-8523 Japan; 4 Leading Medical Research Core Unit, Nagasaki University Graduate School of Biomedical Sciences, Nagasaki, 852-8523 Japan; 5 Division of Strategic Collaborative Research, Atomic Bomb Disease Institute, Nagasaki University, Nagasaki, 852-8523 Japan; 6 Nagasaki University Health Center, Nagasaki, 852-8523 Japan; 7 Department of Public Health, Nagasaki University Graduate School of Biomedical Sciences, Nagasaki, 852-8523 Japan; 8 Department of Island and Community Medicine, Nagasaki University Graduate School of Biomedical Sciences, Nagasaki, 853-0031 Japan

**Keywords:** Diagnostic markers, Predictive markers, Biomarkers, Epidemiology, Preclinical research

## Abstract

Although fast eating speed has been associated with cardiovascular risk factors, no studies have reported an association between fast eating speed and atherosclerosis as evaluated by carotid intima–media thickness (CIMT). Rapid glucose ingestion is known to cause glucose spikes, which may accelerate atherogenesis and increase levels of growth differentiation factor 15 (GDF-15). Therefore, GDF-15 levels may influence the association between fast eating speed and atherosclerosis. To evaluate the association between eating speed and atherosclerosis in relation to GDF-15, this cross-sectional study analyzed 742 Japanese aged 60–69 years. They were required to have normal thyroid hormone levels, because both GDF-15 levels and atherosclerosis (CIMT ≥ 1.1 mm) can be influenced by thyroid dysfunction. Participants were stratified by the median GDF-15 level. A significant positive association was observed between fast eating speed and atherosclerosis, but only among participants with a high GDF-15 level: the sex- and age-adjusted odds ratios (95% confidence intervals) were 1.95 (1.09, 3.48) in participants with a high GDF-15 level, and 0.83 (0.37, 1.88) in those with a low GDF-15 level. This association remained even after further adjustment for thyroid function and metabolic factors. Serum concentrations of GDF-15 may mediate the association between fast eating speed and atherosclerosis.

## Introduction

Self-reported fast eating speed has been reported to be associated with cardiovascular risk factors^1^. Carotid intima–media thickness (CIMT) is a known surrogate marker of atherosclerosis^2^ and an established risk factor for cardiovascular disease^3^.

In addition, self-reported fast eating was revealed as an independent risk factor for height loss among non-overweight Japanese workers^4^. Since height loss was reported to be associated both with cardiovascular disease^5^ and with atherosclerosis as evaluated by CIMT^6^, eating speed could be associated with atherosclerosis.

However, no studies have reported an association between fast eating speed and atherosclerosis as evaluated by CIMT.

Fast eating speed may cause glucose spikes^7^, and swings in blood glucose levels were reported to accelerate atherogenesis in a rat model^8^. Therefore, fast eating speed may be positively associated with atherosclerosis as determined by CIMT, perhaps because habitual glucose spikes accelerate CIMT progression^9^.

Growth differentiation factor 15 (GDF-15) is a stress-responsive member of the transforming growth factor-β (TGF-β) family of cytokines. Since high glucose levels and insulin peaks upregulate GDF-15 transcription^10^, and repeated glucose spikes and insulin resistance might deteriorate endothelial function^8^, GDF-15 levels could influence the association between eating speed and atherosclerosis. Therefore, we hypothesized that fast eating speed would be significantly positively associated with atherosclerosis but only in people with high GDF-15 levels.

Even among euthyroid individuals, thyroid function can influence the development of atherosclerosis^11^. In addition, thyroid function may influence serum GDF-15 levels by regulating energy balance^12^. Therefore, thyroid function may act as a strong confounder regarding the role of GDF-15 in the association between eating speed and atherosclerosis.

Furthermore, GDF-15 is a potential biomarker of aging^13^, while CIMT is known to be significantly associated with age^14^. Thus, to reduce the influences of abnormal thyroid function and age, this cross-sectional study was conducted in participants with a normal thyroid hormone range and a narrow age range of 60–69 years, with the goal of evaluating the association between fast eating speed and atherosclerosis in relation to GDF-15 levels.

## Materials and methods

### Study population

In addition to the annual health check-up recommended by the Japanese government, we conducted our own survey^15^. According to an estimate by the National Institute of Population and Social Security Research, in 2015, the total number of residents in Saza aged 60–69 years was 2103^16^.

The study population comprised 816 Japanese aged 60–69 years from Saza town in western Japan who underwent an annual medical examination in 2014.

Thyroid hormone is known to regulate energy balance in daily life, and GDF-15 is also associated with energy balance^17^. Therefore, since an abnormal thyroid hormone range can cause energy balance dysregulation that could confound the present analysis, all participants were required to have normal ranges of both free triiodothyronine (T3) and free thyroxine (T4). Individuals with a history of thyroid disease (n = 30), those without thyroid hormone data (n = 9) or thyroid-stimulating hormone (TSH) data (n = 2), and those with abnormal thyroid hormone ranges (n = 32) were excluded. Participants without body mass index (BMI) data (n = 1) were also excluded. The remaining 742 participants (300 men and 442 women), with a mean age of 65.1 years (standard deviation (SD), 2.6 years; range, 60–69 years), were included in the study.

Written informed consent forms were used to ensure that participants understood the objectives of the study. All procedures involving human participants were performed in accordance with the ethical standards of our institution’s research committee and with the 1964 Helsinki Declaration and its later amendments for comparable ethical standards. This study was approved by the ethics committee of the Nagasaki University Graduate School of Biomedical Sciences (project registration number 14051404-13).

### Data collection and laboratory measurements

The general methods used in the present study, including thyroid function evaluation, have been described elsewhere^18,19^.

Trained interviewers obtained information on clinical characteristics, specifically history of thyroid disease, eating speed, and medications for hyperglycemia and hyperlipidemia. The self-reported eating rate has been shown to correlate with the objectively measured eating rate^20^. In the present study, participants were asked the following question: “Compared to others, how fast do you eat?” Possible answers were “slow,” “moderate,” and “fast”. Both “slow” and “moderate” were defined as “non-fast eating speed,” while “fast” was defined as “fast eating speed.”

Body weight and height were measured using an automatic body composition analyzer (BF-220; Tanita, Tokyo, Japan). BMI (kg/m^2^) was calculated. A trained observer used a blood pressure measuring device (HEM-907; Omron, Kyoto, Japan) to record the blood pressure in the right arm after the patient rested in a sitting position for at least 5 min. Hypertension was defined as systolic blood pressure ≥ 140 mmHg and/or diastolic blood pressure ≥ 90 mmHg and/or taking anti-hypertensive medication.

To measure GDF-15, serum samples were diluted tenfold with specific MILLIPLEX diluents. GDF-15 concentrations were then determined using a bead-based multiplexed immunoassay system with Luminex xMAP technology. Fasting blood samples were collected. TSH, free T3, and free T4 levels were measured using standard procedures by the LSI Medience Corporation (Tokyo, Japan). Glycohemoglobin (HbA1c), triglyceride (TG), high-density lipoprotein cholesterol (HDLc), and creatinine levels were measured using standard procedures by SRL Inc. (Tokyo, Japan).

An experienced vascular examiner measured both left- and right-sided CIMT of the common carotid arteries using a LOGIQ Book XP with a 10-MHz transducer (GE Healthcare, Milwaukee, WI, USA). Since maximum CIMT values can predict atherosclerotic disease more accurately than mean values^21,22^, the maximum values for the left and right CIMT were then calculated with semi-automated digital edge-detection software (IntimaScope; MediaCross, Tokyo, Japan), using a protocol^23^ described in our previous studies^6,15,18,24^. The recently developed IntimaScope software was used to increase the accuracy and reproducibility of CIMT measurement values. This software semi-automatically recognizes the edges of the internal and external membranes of the artery and automatically determines the distance at a sub-pixel level (estimated to be 0.01 mm)^25^. Since continuous CIMT values never indicate continuous values reflecting atherosclerotic status^26,27^, dichotomized CIMT values were used. Atherosclerosis was diagnosed as a CIMT of ≥ 1.1 mm, because the normal CIMT value was previously reported to be < 1.1 mm^28^.

### Statistical analysis

To validate the study population in the present study, goodness of fit was evaluated using the Hosmer–Lemeshow test. Ages and free T3 and free T4 levels of participants in relation to GDF-15 levels and fast eating speed status are expressed as mean ± SD. Since GDF-15 and TSH levels showed skewed distributions, they are expressed as medians (interquartile range). Prevalences are expressed as percentages. GDF-15 levels are categorized as “low” and “high” according to sex-specific median values: 0.97 ng/mL for men and 0.79 ng/mL for women.

Logistic regression models were used to calculate odds ratios (ORs) and 95% confidence intervals (CIs) to determine the association between GDF-15 levels and atherosclerosis. Logistic regression models were also used to evaluate the association between eating speed status (non-fast eating speed and fast eating speed) and atherosclerosis stratified by GDF-15 levels.

Factors that influence both glucose metabolism and atherosclerosis development could have acted as confounders in the present analysis. Thyroid function, which can impact glucose metabolism^29^, may affect the development of atherosclerosis even in euthyroid individuals^11^. TSH and free T3 may have acted as confounders in the present study. Improvement of glucose metabolism and insulin sensitivity can reduce blood pressure in hypertensive obese participants^30^. Hypertension, which is strongly associated with atherosclerosis^31^, is also associated with insulin resistance^32^. Diabetes with high TG and low HDL has also been shown to be associated with atherosclerosis^33^, and high TG and low HDL indicate strong insulin resistance^34^. Therefore, high BMI, hypertension, high HbA1c, high TG, low HDLc, and medications associated with these factors might have acted as confounders in the present analysis. At the same time, these factors could mediate the association between thyroid function and atherosclerosis.

Three different models were used to adjust for confounding factors. The first model (Model 1) adjusted only for sex and age. The next model (Model 2) adjusted for sex and age, as well as for thyroid-related hormones, namely TSH (logarithmic value) and free T3 (pg/mL). The last model (Model 3) adjusted for sex, age, and known metabolic factors such as high BMI (≥ 25 kg/m^2^), hypertension (yes, no), high HbA1c (≥ 6.5%), high TG (≥ 150 mg/dL), low HDLc (< 40 mg/dL), and taking antihyperglycemic or antihyperlipidemic medications. Dichotomous values (yes, no) were used to categorize the known metabolic factors.

All statistical analyses were performed with SAS for Windows (version 9.4; SAS Inc., Cary, NC, USA). All p-values for statistical tests were two-tailed. All p-values < 0.05 were regarded as statistically significant, except for those evaluating interaction. p-values < 0.2 were regarded as statistically significant for evaluating interaction, based on previous reports^35,36^.

## Results

Among the 742 study participants aged 60–69 years with normal ranges of thyroid hormone, 216 were fast eaters and 96 had atherosclerosis.

### Characteristics of the study population in relation to GDF-15 level

Table 1 shows the characteristics of the study population by GDF-15 level. Compared to participants with a low GDF-15 level, those with a high GDF-15 level were significantly older, had a significantly higher prevalence of hypertension, and had significantly higher BMI and HbA1c.

**Table 1:** Characteristics of study population in relation to serum growth differentiation factor 15 (GDF-15) levels.

	Serum growth differentiation factor 15 (GDF-15) levels		p
	(Low)	(High)	
No. of participants	368	374	
Men, %	40.5	40.4	0.932
Age	64.7 ± 2.7	65.6 ± 2.5	< 0.001
GDF-15, ng/mL	0.69 [0.59, 0.77]^*1^	1.06 [0.94, 1.22]^*1^	< 0.001^*2^
TSH, (0.39–4.01) μIU/mL	1.54 [1.10, 2.28]^*1^	1.58 [1.11, 2.38]^*1^	0.876^*2^
Free T3, (2.1–4.1) pg/mL	3.2 ± 0.3	3.2 ± 0.3	0.450
Free T4, (1.0–1.7) ng/dL	1.3 ± 0.2	1.3 ± 0.2	0.982
High BMI, %	18.5	28.3	0.002
Hypertension, %	45.1	55.3	0.005
High HbA1c, %	5.7	10.4	0.018
High TG, %	13.9	17.4	0.187
Low HDLc, %	5.2	7.2	0.246
Glucose lowering medication, %	5.7	8.8	0.103
Lipid lowering medication, %	28.0	27.5	0.892

TSH, thyroid stimulating hormone; T3, triiodothyronine; T4, thyroxine; BMI, body mass index; HbA1c, glycohemoglobin; TG, triglycerides; HDLc, high-density lipoprotein cholesterol. The normal range is given in parentheses. Values of age, free T3, and free T4 are mean ± standard deviation.

^*1^Values are median [the first quartile, the third quartile].

^*2^Logarithmic transformation was used for evaluating p. Among men, the low GDF-15 level was < 0.97 ng/mL and the high GDF-15 level was ≥ 0.97 ng/mL; among women, the corresponding values were < 0.79 ng/mL and ≥ 0.79 ng/mL, respectively.

### Characteristics of study population in relation to eating speed status

Table 2 shows the characteristics of the study population by eating speed status. Participants who were fast eaters were significantly younger and had a significantly higher BMI than those who were not.

**Table 2:** Characteristics of study population in relation to status of fast eat.

	Fast eating speed		p
	(−)	(+)	
No. of participants	526	216	
Men, %	38.4	45.4	0.079
Age	65.3 ± 2.6	64.6 ± 2.5	< 0.001
GDF-15, ng/mL	0.86 [0.69, 1.06]^*1^	0.87 [0.67, 1.07]^*1^	0.776^*2^
TSH, (0.39–4.01) μIU/mL	1.58 [1.11, 2.32]^*1^	1.50 [1.09, 2.32]^*1^	0.885^*2^
Free T3, (2.1–4.1) pg/mL	3.2 ± 0.3	3.2 ± 0.3	0.781
Free T4, (1.0–1.7) ng/dL	1.3 ± 0.2	1.3 ± 0.2	0.608
High BMI, %	19.4	33.3	< 0.001
Hypertension, %	49.2	52.8	0.382
High HbA1c, %	7.0	10.6	0.101
High TG, %	15.6	15.7	0.959
Low HDLc, %	5.5	7.9	0.227
Glucose lowering medication, %	6.5	9.3	0.184
Lipid lowering medication, %	27.6	28.2	0.853

TSH, thyroid stimulating hormone; T3, triiodothyronine; T4, thyroxine; BMI, body mass index; HbA1c, glycohemoglobin; TG, triglycerides; HDLc, high-density lipoprotein cholesterol. The normal range is given in parentheses. Values of age, free T3, and free T4 are mean ± standard deviation.

^*1^Values are median [the first quartile, the third quartile].

^*2^Logarithmic transformation was used for evaluating p.

### Characteristics of fast eaters by GDF-15 level

Among participants with a high GDF-15 level, fast eaters were younger and had a higher prevalence of elevated BMI compared with non-fast eaters. No significant differences between fast and non-fast eaters were observed among participants with a low GDF-15 level (Table 3).

**Table 3:** Participant characteristics stratified by eating speed and serum growth differentiation factor 15 (GDF-15) levels.

	Serum growth differentiation factor 15 (GDF-15) levels					
	(Low)			(High)		
	Fast eating speed		p	Fast eating speed		p
	(−)	(+)		(−)	(+)	
No. of participants	260	108		266	108	
Men, %	37.3	48.1	0.054	39.5	42.6	0.579
Age	64.8 ± 2.7	64.3 ± 2.5	0.059	65.8 ± 2.5	65.0 ± 2.5	0.004
GDF-15, ng/mL	0.67 [0.58, 0.77]^*1^	1.06 [0.93, 1.21]^*1^	0.223^*2^	1.06 [0.93, 1.21]^*1^	1.07 [0.97, 1.25]^*1^	0.536^*2^
TSH, (0.39–4.01) μIU/mL	1.62 [1.17, 2.24]^*1^	1.38 [1.01, 2.31]^*1^	0.394^*2^	1.56 [1.08, 2.45]^*1^	1.59 [1.21, 2.34]^*1^	0.553^*2^
Free T3, (2.1–4.1) pg/mL	3.2 ± 0.3	3.2 ± 0.3	0.591	3.2 ± 0.3	3.2 ± 0.3	0.919
Free T4, (1.0–1.7) ng/dL	1.2 ± 0.2	1.3 ± 0.1	0.623	1.2 ± 0.2	1.3 ± 0.2	0.811
High BMI, %	16.9	22.2	0.234	21.8	44.4	< 0.001
Hypertension, %	44.6	46.3	0.769	53.8	59.3	0.334
High HbA1c, %	5.8	5.6	0.936	8.3	15.7	0.032
High TG, %	13.8	13.9	0.991	17.3	17.6	0.945
Low HDLc, %	4.6	6.5	0.463	6.4	9.3	0.333
Glucose-lowering medication, %	6.2	4.6	0.567	6.8	13.9	0.028
Lipid-lowering medication, %	27.7	28.7	0.845	27.4	27.8	0.948

TSH, thyroid-stimulating hormone; T3, triiodothyronine; T4, thyroxine; BMI, body mass index; HbA1c, glycohemoglobin; TG, triglycerides; HDLc, high-density lipoprotein cholesterol. The normal range is given in parentheses. Values of age, free T3, and free T4 are mean ± standard deviation.

^*1^Values are median [first quartile, third quartile].

^*2^Logarithmic transformation was used to evaluate p. Among men, the low GDF-15 level was < 0.97 ng/mL and the high GDF-15 level was ≥ 0.97 ng/mL; among women, the corresponding values were < 0.79 ng/mL and ≥ 0.79 ng/mL, respectively.

### Association between GDF-15 level and atherosclerosis

Table 4 shows the ORs (95%CIs) of atherosclerosis by GDF-15 level. Compared to the participants with low GDF-15, those with high GDF-15 had significantly higher ORs of atherosclerosis in the sex- and age-adjusted model (model 1), thyroid hormone–adjusted model (model 2), and metabolic factor–adjusted model (model 3).

**Table 4:** Association between growth differentiation factor 15 (GDF-15) levels and atherosclerosis.

	Serum growth differentiation factor 15 (GDF-15) levels		*p*
	(Low)	(High)	
	368	374	
	35 (9.5)	61 (16.3)	
Model 1	Reference	1.76 (1.12, 2.77)	0.014
Model 2	Reference	1.78 (1.13, 2.80)	0.012
Model 3	Reference	1.68 (1.06, 2.67)	0.026

Model 1: adjusted only for sex and age. Model 2: in addition to sex and age, adjusted further for thyroid-stimulating hormone (TSH) and free triiodothyronine (T3). Model 3: in addition to sex and age, adjusted further for hypertension, high body mass index (BMI), high hemoglobin A1c (HbA1c), low high-density lipoprotein cholesterol (HDLc), high triglycerides (TG), glucose-lowering medication, and anti-hyperlipidemic medication. Among men, the low GDF-15 level was < 0.97 ng/mL and the high GDF-15 level was ≥ 0.97 ng/mL; among women, the corresponding values were < 0.79 ng/mL and ≥ 0.79 ng/mL, respectively.

### Association between fast eating speed and atherosclerosis with stratification by GDF-15 level

The associations between fast eating speed and atherosclerosis, with stratification by GDF-15 level, are shown in Table 5. In the high GDF-15 group only, fast eating speed was significantly positively associated with atherosclerosis. The sex- and age-adjusted ORs (95%CIs) of atherosclerosis were 1.95 (1.09, 3.48) for high GDF-15 and 0.83 (0.37, 1.88) for low GDF-15. These associations remained even after further adjustment for thyroid hormones (model 2) and for known metabolic factors (model 3). In model 2, the adjusted ORs (95%CIs) of atherosclerosis were 1.93 (1.08, 3.46) for high GDF-15 and 0.84 (0.36, 1.92) for low GDF-15, while in model 3, the corresponding values were 2.07 (1.12, 3.82) and 0.82 (0.35, 1.87).

**Table 5:** Association between fast eating speed and atherosclerosis by growth differentiation factor 15 (GDF-15) levels.

	Serum growth differentiation factor 15 (GDF-15) levels						Interaction
	(Low)			(High)			
	Fast eating speed		*p*	Fast eating speed		*p*	
	(−)	(+)		(−)	(+)		
	260	108		266	108		
	26 (10.0)	9 (8.3)		36 (13.5)	25 (23.1)		
Model 1	Reference	0.83 (0.37, 1.88)	0.661	Reference	1.95 (1.09, 3.48)	0.024	0.060
Model 2	Reference	0.84 (0.36, 1.92)	0.674	Reference	1.93 (1.08, 3.46)	0.027	0.065
Model 3	Reference	0.82 (0.35, 1.87)	0.630	Reference	2.07 (1.12, 3.82)	0.020	0.028

Model 1: adjusted only for sex and age. Model 2: in addition to sex and age, adjusted further for thyroid-stimulating hormone (TSH) and free triiodothyronine (T3). Model 3: in addition to sex and age, adjusted further for high body mass index (BMI), hypertension, high hemoglobin A1c (HbA1c), low high-density lipoprotein cholesterol (HDLc), high triglycerides (TG), glucose-lowering medication, and anti-hyperlipidemic medication. Among men, the low GDF-15 level was < 0.97 ng/mL and the high GDF-15 level was ≥ 0.97 ng/mL; among women, the corresponding values were < 0.79 ng/mL and ≥ 0.79 ng/mL, respectively.

### Effect of interaction of GDF-15 level on the association between fast eating speed and atherosclerosis

Since p < 0.2 for interaction is considered to be statistically significant^35,36^, this study demonstrated a significant interaction of GDF-15 level on the association between fast eating speed and atherosclerosis (Table 5). The p values of the interaction were 0.060 for model 1, 0.065 for model 2, and 0.028 for model 3.

### White blood cell count stratified by eating speed and GDF-15 level

Five hundred twelve participants had data on white blood cell count. Among those with a low GDF-15 level, fast eaters had a significantly higher white blood cell count than non-fast eaters. No significant difference was observed among those with a high GDF-15 level. Among those with a low GDF-15 level, the mean ± SD of the white blood cell count was 5561 ± 1290 cells/µL in fast eaters (n = 61) and 5063 ± 1290 cells/µL in non-fast eaters (n = 172) (p = 0.007). Among those with a high GDF-15 level, the corresponding values were 5525 ± 1267 cells/µL in fast eaters (n = 80) and 5633 ± 1405 cells/µL in non-fast eaters (n = 199) (p = 0.551).

## Discussion

The major finding in this study of participants with normal thyroid hormone ranges was that fast eating speed was positively associated with atherosclerosis, but only in individuals with a high GDF-15 level.

Previous studies showed that fast eating speed was significantly positively associated with components of metabolic syndrome^1,37^, especially hypertriglyceridemia^1^. A study of 30 healthy participants reported an association between the ingestion speed of a sugary beverage and the rapid elevation of blood glucose level^7^. Therefore, habitually fast eating speeds may induce repeated glucose spikes in daily life. Since such spikes impaired endothelium-dependent aortic relaxation in a rat model^8^, and frequent fluctuations in blood glucose accelerated atherogenesis in a mouse model^38^, fast eating speed may be associated with atherosclerosis. However, no cohort study has revealed a positive association between fast eating speed and atherosclerosis as evaluated by CIMT.

In the present study, fast eating speed was significantly positively associated with atherosclerosis, but only in participants with a high GDF-15 level. However, the mechanism underlying this association is unknown. Since GDF-15 levels were essentially the same in participants with fast or non-fast eating speeds (Table 2), GDF-15 levels may not be strongly increased by fast eating speed itself, but rather by glucose spike frequency, which is related to fast eating speed^7,10^. Therefore, the glucose spike frequency associated with fast eating speed may be higher in individuals with elevated GDF-15 level than in those without. Also, because repeated glucose spikes may impair endothelial function^8^, the combination of high GDF-15 levels and fast eating speed might cause the development of atherosclerosis.

In the present study, fast eating speed was associated with a significantly higher prevalence of high BMI and high HbA1c than non-fast eating speed, but only in participants with a high GDF-15 level. Self-reported fast eating speed has been shown to be associated with high BMI^39^ and diabetes^40^. Since high glucose levels and insulin peaks upregulate GDF-15 transcription^10^, physical conditions that elevate GDF-15 levels due to fast eating might be associated with high BMI and HbA1c levels. However, the present study revealed that high BMI and HbA1c levels could not act as substitute factors to eating speed. Even after adjusting for high levels of both, a significant positive association between fast easting speed and atherosclerosis was observed only in participants with a high GDF-15 level. Furthermore, in our additional analyses, high BMI and HbA1c levels were not significantly associated with atherosclerosis regardless of whether the GDF-15 level was high or low. Among participants with a low GDF-15 level, the sex- and age-adjusted (model 1) ORs (95%CI) for atherosclerosis were 1.69 (0.74, 3.86) for high BMI and 2.09 (0.64, 6.82) for high HbA1c. Among participants with a high GDF-15 level, the corresponding values were 0.84 (0.45, 1.57) and 1.38 (0.60, 3.18).

Cells of the monocyte/macrophage lineage may have played a crucial role in the present results. Although GDF-15 itself was shown to inhibit the development of atherosclerosis^41^, previous studies indicated a positive association between GDF-15 and atherosclerosis^24,42,43^. This association is compatible with the results of the present study showing that participants with a high GDF-15 level had significantly higher ORs of atherosclerosis than those with a low GDF-15 level (Table 4). Macrophage activity might contribute to this positive association.

Macrophages are fundamentally involved in the development of atherosclerotic lesions^44^. GDF-15 involves in macrophage activity^45^. GDF-15 is produced by activated macrophages^46,47^. Therefore, individuals with higher levels of serum GDF-15 might have greater macrophage activity, indicating a higher risk of developing atherosclerosis. In the present study, only participants with a high GDF-15 level exhibited a significant positive association between fast eating speed and atherosclerosis (Table 5). Thus, high GDF-15 levels might amplify the influence of fast eating speed on atherosclerosis development.

Increased activity of monocyte/macrophage lineage cells^48,49^ plays a crucial role in the development of atherosclerosis. Repetitive fluctuations in blood glucose levels might induce monocyte adhesion to the endothelium^50^. Therefore, if there is greater activity of monocyte/macrophage lineage cells due to high GDF-15 levels, fast eating speed may be positively associated with atherosclerosis by indicating the presence of habitual glucose spikes.

We also evaluated the white blood cell count in the study population, since it may reflect inflammatory activity. Fast eating was associated with high odds of atherosclerosis but only among participants with a high GDF-15 level. Conversely, fast eaters had a significantly higher white cell count than non-fast eaters, but only among participants with a low GDF-15 level. Therefore, the association between the white blood cell count and inflammatory activity might not be the best explanation for the present results. Since an inconsistent correlation was reported between the white cell count and carotid atherosclerosis^51,52^, clarifying the influence of GDF-15 on atherosclerosis progression might identify a novel mechanism underlying atherosclerosis progression.

This is the first study to reveal a positive association between fast eating speed and atherosclerosis in a general population, and to demonstrate the influence of GDF-15 level on this correlation. Therefore, measuring GDF-15 levels may be an efficient means of evaluating atherosclerosis risk as related to eating speed. While further investigation is necessary, the present findings clarify the mechanisms underlying the link between eating speed and the development of atherosclerosis.

Potential limitations of this study warrant consideration. The exact cutoff for the GDF-15 level that determines the association between fast eating speed and atherosclerosis in the general population is unknown. Since GDF-15 was reported to be a marker of aging^13^, identifying the GDF-15 cutoff point should take age into consideration, and a larger study that enables us to perform stratification by age groups is necessary. Since we had no data on glucose spikes in this study population, we could not evaluate the influence of these spikes on the present results. Finally, this was a cross-sectional study and therefore causal relationships could not be established.

## Conclusion

In conclusion, in a general elderly population, GDF-15 level was a determinant of the association between fast eating speed and atherosclerosis. This indicates that a novel mechanism underlies the association between eating speed and the development of atherosclerosis.

## Data Availability

We cannot publicly provide individual data due to participant privacy, according to ethical guidelines in Japan. Additionally, the informed consent was obtained does not include a provision for publicity sharing data. Qualifying researchers may apply to access a minimal dataset by contacting Prof Naomi Hayashida, Principal Investigator, Division of Promotion of Collaborative Research on Radiation and Environment Health Effects, Atomic Bomb Disease Institute, Nagasaki University, Nagasaki, Japan at naomin@nagasaki-u.ac.jp. Or, please contact the office of data management at ritouken@vc.fctv-net.jp. Information for where data request is also available at https://www.genken.nagasaki-u.ac.jp/dscr/message/ (accessed on 23 May 2023) and http://www.med.nagasaki-u.ac.jp/cm/ (accessed on 23 May 2023).
